# The Development and Evaluation of an SYBR Green I-Based qPCR Assay for Detecting the Marek’s Disease Virus SC9-1 Vaccine Strain

**DOI:** 10.3390/v18070717

**Published:** 2026-06-29

**Authors:** Ruihan Shi, Haijun Jiang, Chang Liu, Mengzhu Dong, Tingwen Zheng, Rujun Ye, Yu Zhou

**Affiliations:** Institute of Animal Husbandry and Veterinary Medicine, Beijing Academy of Agriculture and Forestry Sciences, Beijing 100097, China

**Keywords:** Marek’s disease virus, SC9-1 vaccine strain, real-time quantitative PCR, SYBR Green I

## Abstract

The increasing virulence of field strains of Marek’s disease virus (MDV), which can overcome the immunity conferred by the widely used CVI988 vaccine, underscores the need for more effective alternatives. The SC9-1 vaccine strain has demonstrated superior protective efficacy and is being increasingly adopted in China. Importantly, it harbors a unique recombinant REV-LTR insertion junction that is absent from all other MDV strains. Targeting this strain-exclusive genomic locus, this study established an SYBR Green I-based quantitative real-time PCR assay for specific detection and quantification of SC9-1 to support clinical vaccine surveillance. We designed specific primers and systematically optimized the qPCR reaction conditions. The resulting assay demonstrated high specificity, showing no cross-reactivity with other MDV strains or common avian pathogens. It achieved a sensitivity of 1.0 × 10^1^ copies/μL, and reproducibility was excellent, with both intra- and inter-assay coefficients of variation below 1.0%. Furthermore, the LOD of this assay for commercial SC9-1 vaccine samples was determined to be 1.22 PFU per 200 μL sample. In conclusion, this SYBR Green I-based qPCR assay provides a specific, sensitive, and reproducible tool for the rapid quantification of the SC9-1 vaccine virus. It is fully validated for routine vaccine quality control, while exhibiting promising potential for subsequent field surveillance within MD vaccination programs.

## 1. Introduction

Marek’s disease, caused by MDV, is a lymphoproliferative neoplastic disorder of chickens that causes substantial economic losses in the global poultry industry annually [1,2,3]. Vaccination remains the cornerstone strategy for MD control. The naturally attenuated CVI988/Rispens vaccine strain is used worldwide [4,5,6,7,8]. However, continuous evolution of field virus virulence has led to the emergence of strains capable of breaking through the immune protection conferred by the CVI988/Rispens vaccine [9,10,11,12]. The SC9-1 strain is a genetically engineered deletion vaccine developed in China. It is known for its safety and strong immunogenicity [13]. Studies have demonstrated that the SC9-1 vaccine provides superior protection against prevalent MDV field strains compared to CVI988/Rispens [13,14]. It is now extensively used in high-MD-incidence areas of China, like Guangdong, Guangxi, Yunnan, and Fujian.

Accurate monitoring of MD vaccine virus replication in immunized flocks is important for evaluating vaccination efficacy in clinical practice [15,16]. Currently, the primary approaches for MDV detection include virus isolation, conventional PCR coupled with gel electrophoresis, and real-time qPCR [17]. Recently, a multiplex PCR (mPCR) method targeting the *meq* and *gB* genes has been established to simultaneously differentiate MDV-1, MDV-2, and HVT strains, providing a convenient tool for routine clinical diagnosis [18]. Real-time qPCR has also been widely applied to dynamically monitor viral load changes in various tissues, especially feather tips (the follicle-bearing feather base), which serve as ideal non-invasive samples for evaluating vaccine replication and immune efficacy [19]. Nevertheless, the end-point conventional PCR method is limited in precise quantification and cannot meet the requirement for dynamic monitoring of vaccine replication in vivo. Furthermore, most existing qPCR assays for MDV are designed either for the broad-spectrum detection of serotype 1 strains or for the specific identification of conventional vaccine strains, such as CVI988/Rispens or herpesvirus of turkeys (HVT) [15,20,21]. Consequently, these methods lack the specificity required to discriminate the SC9-1 vaccine strain from wild-type MDV strains and other MDV vaccine strains (Table S1).

The SC9-1 vaccine was constructed by knocking out the oncogenic *meq* gene from its parental strain GX0101 (GenBank: JX844666.1). The parental virus naturally acquired a reticuloendotheliosis virus long terminal repeat (REV-LTR) fragment through recombination; this chimeric insertion is unique to SC9-1 and genetically stable after serial viral passages (Table S2, Figure S1, Data S1). SYBR Green I-based qPCR offers considerable advantages including operational simplicity, cost-effectiveness, and broad applicability, thereby representing a robust platform for the detection and quantification of diverse pathogens [22,23,24,25,26,27]. In this study, we established an SYBR Green I-based qPCR assay with primers targeting the chimeric junction region of the REV-LTR insertion. This design enables exclusive identification of the SC9-1 vaccine strain. The assay was validated using vaccine strain samples and showed excellent performance for SC9-1 quantification. This newly developed method lays a foundation for subsequent clinical application, and has the potential to serve as a reliable tool for vaccine monitoring and quality assessment in poultry production.

## 2. Materials and Methods

### 2.1. Virus Strains and Nucleic Acids

Vaccine strains MDV SC9-1 and CVI988/Rispens were provided by Beijing Lingyu Biotechnology Co., Ltd., Beijing, China, sourced from their commercial live MD vaccines. The seed passage information of these vaccines is confidential and cannot be provided. HVT and 814 MDV vaccine strains were purchased from Harbin Pharmaceutical Group Animal Health Co., Ltd. (Harbin, China), with undisclosed seed passage history. Nucleic acids of virulent MDV-1 Md5, ALV-J and REV were gifted by Prof. Cui Zhizhong and Prof. Zhao Peng (Shandong Agricultural University), without accompanying passage records. Wild MDV isolates WTMDV-1 to WTMDV-7 were recovered from tissue samples of naturally MDV-affected chickens across multiple regions: WTMDV-1 (Zhangzhou, Fujian); WTMDV-2, WTMDV-4 (Yingde, Guangdong); WTMDV-3 (Xiangyun, Yunnan); WTMDV-5, WTMDV-6 (Qinzhou, Guangxi Zhuang Autonomous Region); and WTMDV-7 (Heyuan, Guangdong). Their nucleic acids are archived in our laboratory, and post-isolation passage data are unavailable. Nucleic acid stocks of CIAV, NDV and IBDV were preserved in our laboratory, and full serial passage records for these pathogens were not retained.

### 2.2. Reagents and Instruments

The Viral Nucleic Acid Extraction Kit, SYBR Premix ExTaq qPCR Kit, and pCE2-T Vector were purchased from Vazyme Biotech Co., Ltd. (Nanjing, China). The Plasmid Extraction Kit and Gel Extraction Kit were obtained from TIANGEN Biotech Co., Ltd. (Beijing, China). *Escherichia coli* DH5α competent cells were purchased from TransGen Biotech Co., Ltd. (Beijing, China). All qPCR reactions were performed using a CFX Duet Real-Time PCR System (Bio-Rad, Hercules, CA, USA).

### 2.3. Primer Design

We amplified and sequenced the target REV-LTR chimeric region from SC9-1 virus subjected to more than 40 serial passages (Figure S1, Data S1). Sequence alignment demonstrated 100% nucleotide identity between high-passage SC9-1 and its parental strain GX0101, verifying the high genetic conservation of this target locus. In the present study, primers were innovatively designed to target the recombinant chimeric region spanning the LTR insertion fragment and the native genomic sequence of SC9-1 using Primer Premier 5.0 (Premier Biosoft, Palo Alto, CA, USA) software. Primer sequences are listed in Table 1. The expected amplicon size was 101 bp. Primers were synthesized by Sangon Biotech (Shanghai, China) Co., Ltd.

### 2.4. Optimization of qPCR Assay

Quantitative real-time PCR was performed in a 20 μL reaction volume consisting of 10 μL SYBR Premix Taq, primers at specified concentrations, 1 μL DNA template, and nuclease-free distilled water (ddH_2_O). Initially, using the same SC9-1 nucleic acid sample as template, qPCR reactions were prepared at a final primer concentration of 0.5 μM. The thermal profile was designed according to standard DNA polymerase amplification protocols. Given the short 101 bp length of the REV-LTR chimeric target fragment, a 10 s extension step was applied. We set 40 amplification cycles to secure adequate detection capacity for low-abundance SC9-1 templates while eliminating fluorescence saturation from over-amplification, which laid a sound basis for consistent quantitative readouts. To further refine reaction performance, we performed a gradient annealing assay to screen the optimal reaction temperature. The full thermal program began with a pre-denaturation step at 95 °C for 30 s, followed by 40 cycles of 95 °C denaturation for 5 s and 30 s annealing at each tested temperature (57.0 °C, 57.3 °C, 58.0 °C, 59.0 °C, 60.1 °C, 61.1 °C, 61.7 °C, 62.0 °C). Melting curve scanning from 65 °C to 95 °C was carried out after amplification. The ideal annealing temperature was selected to produce the strongest fluorescent signal, lowest cycle threshold (Ct) value, and a single sharp melting peak.

After establishing the optimal thermal cycling conditions, qPCR was performed using final primer concentrations of 0.1, 0.2, 0.3, 0.4, 0.5, and 0.6 μM. The optimal primer concentration for the amplification system was determined according to the highest fluorescence signal intensity coupled with the lowest Ct value.

### 2.5. Preparation of Plasmid Standard

Viral DNA from the SC9-1 strain vaccine served as a template for PCR amplification with primers F-SC9-1 and R-SC9-1. The PCR product was gel-purified, cloned into the pCE2-T/A vector, and transformed into E. coli DH5α competent cells. Positive clones were screened and verified by sequencing. The recombinant plasmid was extracted, and its concentration was measured spectrophotometrically. The copy number was calculated using the formula: copies/μL = [concentration (g/μL)/(plasmid length × 660)] × 6.02 × 10^23^. The plasmid was serially diluted by ten-fold from 1.0 × 10^9^ to 1.0 × 10^1^ copies/μL to serve as qPCR standards. A standard curve was generated by plotting the logarithm of the copy number against the corresponding Ct value.

### 2.6. Specificity Test

Specificity was checked using DNA from MDV strains (SC9-1, CVI988/Rispens, 814, Md5, HVT, WTMDV-1 to WTMDV-7) and CIAV, plus cDNA from ALV-J, REV, NDV, and IBDV. The recombinant plasmid was used as positive control. The ddH_2_O served as no-template control (NTC).

### 2.7. Sensitivity Testing

Sensitivity was tested with ten-fold dilutions of the plasmid standard samples (1 × 10^1^ to 1 × 10^9^ copies/μL), with three repeats per dilution. The limit of detection was determined as the lowest concentration with a clear and typical amplification curve.

### 2.8. Repeatability Test

Repeatability was tested with three plasmid concentrations (1 × 10^6^, 1 × 10^7^, and 1 × 10^8^ copies/μL) in triplicate in one run (intra-assay) and across three runs (inter-assay). Repeatability was evaluated by calculating the coefficient of variation (CV) of Ct values.

### 2.9. Detection of Commercial Vaccine Samples

The commercial SC9-1 vaccine was serially diluted 10-fold to generate gradients from 0.122 PFU/200 μL to 1.22 × 10^6^ PFU/200 μL. Uniform nucleic acid extraction and subsequent qPCR assays were performed to evaluate the LOD of this established assay for vaccine samples.

## 3. Results

### 3.1. Results of qPCR Assay Optimization

**Figure 1:**
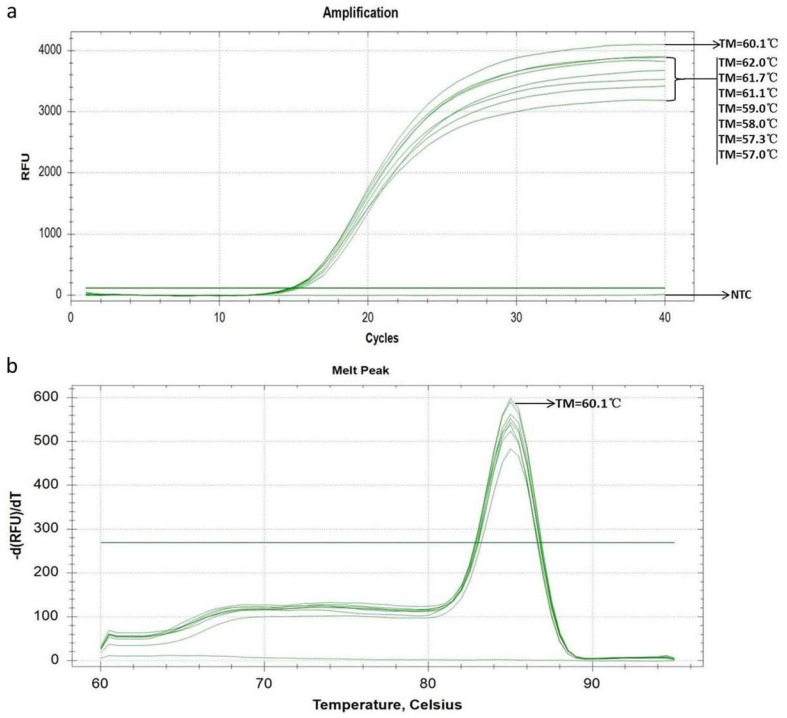
Optimization of annealing temperature for the qPCR thermal cycling protocol. The results indicate that (**a**) the amplification curve achieved the strongest fluorescence intensity and the lowest cycle threshold value at an annealing temperature of 60.1 °C. (**b**) The corresponding melting curve presents a single distinct amplification peak.

The results of the amplification program optimization demonstrated that when the primer annealing temperature was set at 60.1°C, the amplification curve achieved the highest fluorescence intensity and the lowest cycle threshold value, while the melting curve showed a single, distinct amplification peak (Figure 1). These findings indicate that the primer pair achieves the optimal binding efficiency with the template at 60.1 °C. Additionally, no obvious primer dimers were formed during the amplification process. Consequently, the optimized amplification program was determined as follows: initial denaturation at 95 °C for 30 s, followed by 40 cycles of denaturation at 95 °C for 5 s and annealing/extension at 60 °C for 30 s.

**Figure 2:**
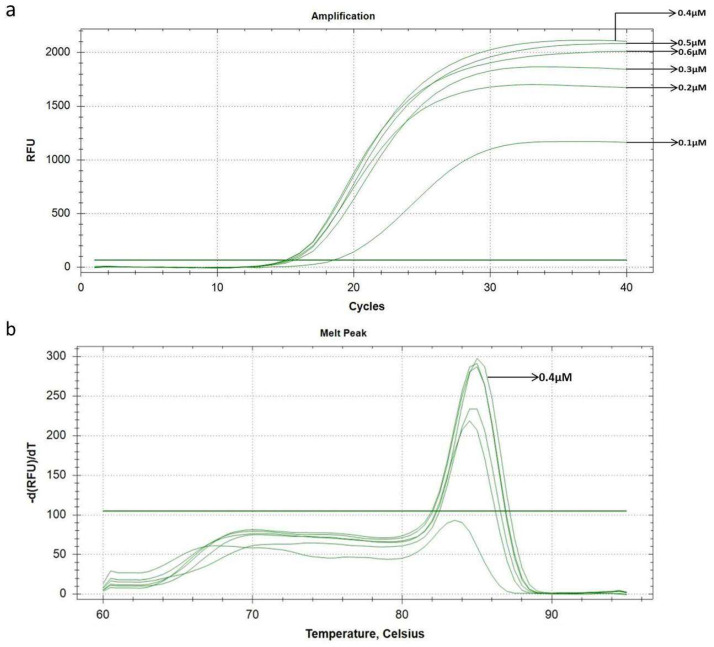
Gradient screening of primer concentrations for qPCR. (**a**) A primer concentration of 0.4 μM yielded high fluorescence intensity and low Ct values; further concentration elevation (0.5–0.6 μM) did not improve amplification performance. (**b**) All tested concentrations generated single melting peaks with no detectable primer dimers.

The results of the gradient primer concentration detection revealed that when the final primer concentration in the amplification system was 0.4 μM, the amplification curve displayed a relatively high fluorescence intensity and a small Ct value (Figure 2a). In contrast, elevating the primer concentration to 0.5 μM or 0.6 μM did not result in a significant improvement in fluorescence intensity or the favorable Ct values observed in the amplification curves. Furthermore, the melting curves of amplicons generated at all tested primer concentrations displayed a single sharp peak, with no obvious formation of primer-dimers (Figure 2b). Therefore, the final primer concentration in the amplification system was determined to be 0.4 μM, and all subsequent experiments were conducted based on this optimized amplification system.

### 3.2. Establishment of Standard Curve

**Figure 3:**
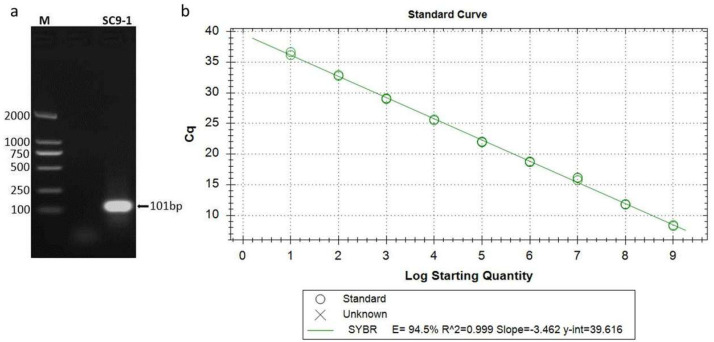
The construction and linear validation of the recombinant plasmid standard. (**a**) A specific 101 bp target fragment was amplified from SC9-1 vaccine DNA, purified, cloned into the pCE2-T/A vector and transformed into *E. coli* DH5α. (**b**) Serial 10-fold dilutions (1.0 × 10^1^–1.0 × 10^9^ copies/μL) of purified plasmid were used to generate a standard curve. The linear regression equation was y = −3.462x + 39.616, with R^2^ = 0.999 and an amplification efficiency of 94.5%.

Viral DNA extracted from the SC9-1 strain vaccine was used as the template for PCR amplification with primers F-SC9-1 and R-SC9-1, generating a specific amplicon of 101 bp (Figure 3a). The positive clone of the recombinant plasmid was screened and verified by sequencing. Plasmid purity and integrity were initially assessed by micro-spectrophotometry, which yielded a single sharp 260 nm absorbance peak free of degradation signals. Agarose electrophoresis further confirmed intact plasmid profiles dominated by supercoiled DNA, with no obvious degradation smearing. A standard curve of the recombinant plasmid was successfully constructed (Figure 3b). The qPCR assay demonstrated a strong linear relationship between Ct value and the logarithm of the template copy number. The equation was generated as y = −3.462x + 39.616. The correlation coefficient (R^2^) was 0.999, showing a good linear fit. The amplification efficiency was 94.5% over 1.0 × 10^1^ to 1.0 × 10^9^ copies/μL.

### 3.3. Specificity and Sensitivity

**Figure 4:**
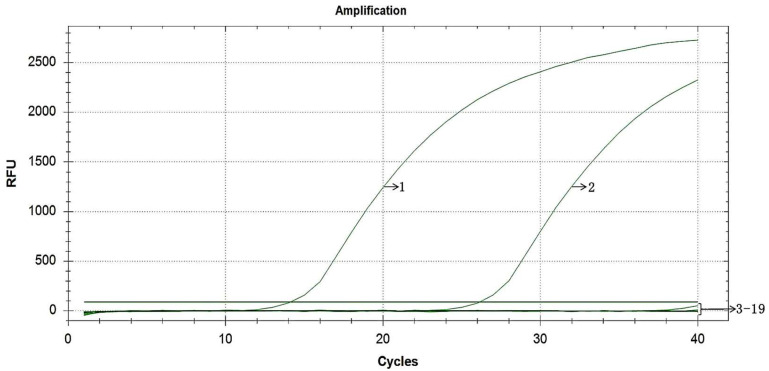
Specificity analysis of the SYBR Green I-based qPCR assay. The established assay specifically amplified DNA from the SC9-1 vaccine strain (curve 1) and its corresponding recombinant plasmid standard (curve 2). No amplification signals were detected from nucleic acids of other MDV strains or common avian pathogens (curves 3–19: CVI988, 814, Md5, HVT, WTMDV-1~WTMDV-7, CIAV, ALV-J, REV, IBDV, NDV and NTC).

This detection method specifically amplified the target fragments of MDV SC9-1 strain and its corresponding plasmid standard (Figure 4). No specific amplification signals were observed in other MDV vaccine strains, standard virulent strains, field-isolated virulent strains and other common avian pathogenic viruses, which verified the strong target discrimination of the established assay.

**Figure 5:**
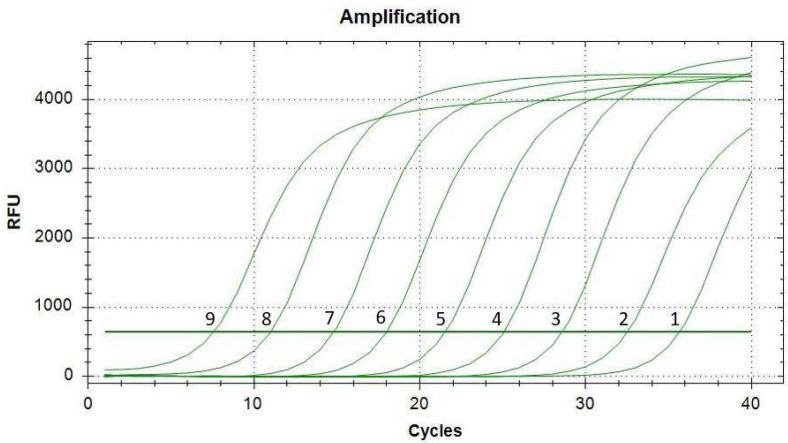
Sensitivity detection of the qPCR assay using serially diluted recombinant plasmid. Curves 1–9 correspond to plasmid concentrations from 1.0 × 10^1^ to 1.0 × 10^9^ copies/μL. The limit of detection (LOD) of this method was determined as 1.0 × 10^1^ copies/μL.

Sensitivity testing determined the LOD of the established qPCR assay was 1.0 × 10^1^ copies/μL, with a clear and typical amplification curve at this level (Figure 5). These results confirm the low detection threshold of the method.

### 3.4. Repeatability

The intra-assay CVs of Ct values were all below 1.0% (0.1975–0.4033%), with mean Ct values ranging from 11.24 to 19.17 (95% CI: 11.13–11.36 to 19.07–19.26). The inter-assay CVs were also less than 1.0% (0.3046–0.7372%), and the mean Ct values ranged from 11.54 to 19.37 (95% CI: 11.33–11.75 to 19.17–19.57). The consistent Ct readings across parallel and independent runs validate stable detection consistency of this qPCR system (Table 2).

### 3.5. Determination of the Limit of Detection for Commercial SC9-1 Vaccine

**Figure 6:**
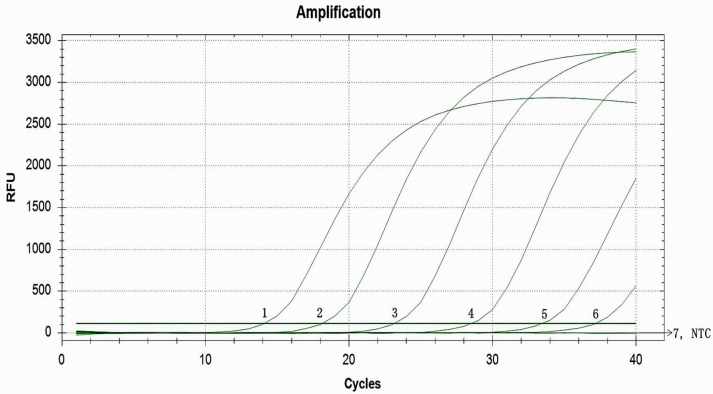
LOD evaluation using serially diluted commercial SC9-1 vaccine samples. Curves 1–6 represent vaccine nucleic acids ranging from 1.22 × 10^6^ PFU/200 μL to 1.22 PFU/200 μL, with Ct values of 14.08, 18.12, 23.14, 28.48, 33.41 and 37.14, respectively. No target amplification was detected at 0.122 PFU/200 μL (curve 7), consistent with the NTC.

The LOD of the established qPCR assay was assessed using commercial SC9-1 vaccine. The results revealed that distinct positive amplification curves could be successfully obtained from nucleic acids extracted from serially diluted vaccine samples within the concentration range of 1.22 PFU/200 μL to 1.22 × 10^6^ PFU/200 μL (Figure 6). In contrast, no specific amplification signal was detected when the vaccine concentration was further reduced to 0.122 PFU/200 μL. Accordingly, the LOD of this qPCR method for detecting commercial SC9-1 vaccine samples was confirmed to be 1.22 PFU per 200 μL diluted vaccine sample.

## 4. Discussion

MDV represents a severe threat to both laying hens and commercial meat-type chickens, causing significant pathogenic and immunosuppressive damage [28,29,30]. Cross-host infections have become increasingly common in turkeys, backyard birds, and ornamental birds [31,32,33]. MDV was first detected in commercial turkeys in Slovenia, showing a very virulent pathotype and high homology to Tunisian chicken strains [31]. In Brazil and Peru, MDV circulates widely in multiple ornamental and backyard avian species, which act as viral reservoirs and bridge transmission between backyard and commercial poultry [33], exacerbating MDV evolution and epidemic risks. Despite widespread vaccination, outbreaks still occur in vaccinated flocks due to the emergence of hypervirulent field strains capable of escaping vaccine-induced immunity, leading to visceral lymphomas, increased mortality, and production losses [34,35]. In recent years, with the advancement of molecular detection techniques, monitoring the replication dynamics of MD vaccine viruses in chickens has emerged as a key research focus for evaluating vaccine immune quality. Studies have demonstrated that early co-infection with oncogenic MDV at 7 days of age does not significantly affect the replication of CVI988, SB-1, and most HVT vaccine strains in feather pulp, and that these MDV vaccines replicate efficiently in feather tips [36,37]. Baigent et al. [15] further confirmed that qPCR-based quantification of CVI988 in feather tips enables reliable validation of successful vaccination and identification of the mechanisms underlying vaccine failure. These findings highlight the great value of qPCR and support feather tips as an ideal non-invasive sample for evaluating vaccine replication, thus allowing for early, rapid, and reliable assessment of flock immune status. Previous studies also indicated that CVI988 viral load in feather tips is significantly affected by immunization dosage, with a clear quantitative correlation between viral replication and protective efficacy against highly virulent MDV challenge [16]. Additionally, Islam et al. [38] verified that real-time qPCR allows for sensitive and robust detection and quantification of CVI988 in broiler house dust, which helps optimize vaccination strategies, reduce disease risk, and alleviate selective pressure for viral virulence evolution. Collectively, these findings highlight the value of qPCR assays for detecting and assessing MDV vaccines.

The emergence of evolving hypervirulent MDV strains capable of breaking through the protection conferred by conventional vaccines such as CVI988/Rispens has posed a major challenge to the poultry industry, highlighting an urgent demand for precise molecular monitoring approaches and novel vaccine development. The SC9-1 vaccine characterized by its demonstrated superior efficacy in high-incidence regions of China represents such an advancement. However, its use has been limited by the lack of a strain-specific quantitative detection method. Existing assays targeting SC9-1 detection, which are largely dependent on conventional PCR, are insufficient for the precise quantification of vaccine replication owing to their qualitative characteristics, inferior sensitivity, and complicated operational procedures [39] (Table S1). To address this need, we developed an SYBR Green I-based qPCR assay for the specific detection and quantification of the SC9-1 strain.

In this study, primers were designed based on the unique recombinant LTR integration region of the SC9-1 genome. Analytical validation confirmed the platform’s exclusive discriminatory capacity: amplification signals were only generated from SC9-1 templates, without off-target amplification from other MDV serotypes or common avian viral pathogens. Sensitivity tests revealed that the LOD was 1.0 × 10^1^ copies/μL for plasmid standards and 1.22 PFU per 200 μL for commercial SC9-1 vaccine samples. The standard curve showed favorable linearity across a wide concentration range, and parallel testing produced highly consistent Ct readouts. All analytical metrics prove this platform a trustworthy quantitative testing system. This qPCR tool could serve to track the in vivo replication kinetics of SC9-1 in vaccinated chickens and facilitate further investigation into the correlation between viral load magnitude and protective immune potency in prospective farm trials. Meanwhile, the closed-tube design of qPCR effectively reduces carryover contamination compared with conventional gel-based PCR. This newly established assay provides a solid technical foundation for subsequent application and aids in optimizing vaccination strategies on farms, thereby improving the overall efficiency of Marek’s disease prevention and control.

It should be acknowledged that this study has several inherent limitations. As an SYBR Green I-based detection system, our assay is cheaper and simpler to implement but suffers from the risk of non-specific amplification and relies on melting curve identification, whereas TaqMan probe qPCR delivers superior specificity; thus this method fits routine SC9-1 vaccine screening in resource-limited facilities. All analytical validations were exclusively conducted using purified plasmid standards and commercial SC9-1 live vaccine preparations. No field clinical samples, co-infected specimens containing both wild-type MDV and SC9-1, or MDV isolates collected from regions outside China were included in the present validation system. Moreover, cross-operator, cross-instrument and inter-laboratory reproducibility tests were not carried out in the current study. To fully characterize the practical performance of this qPCR assay, subsequent trials will conduct systematic field verification on commercial farms with large clinical batches, co-infection specimens and geographically diverse MDV isolates. Parallel testing across different devices and collaborative inter-laboratory trials will also be performed to confirm the platform’s stability before its extensive on-farm deployment.

## 5. Conclusions

In the present study, an SYBR Green I-based qPCR assay was successfully developed for specific detection and precise quantification of the MDV SC9-1 vaccine strain. Comprehensive analytical validation confirmed that this platform features exclusive strain discrimination, low detection thresholds, and stable detection consistency across repeated tests. As a promising technical tool for SC9-1 vaccine management, this method allows for rapid viral quantification, assists in routine vaccine quality evaluation, and provides fundamental support for standardized Marek’s disease prevention and control.

## Figures and Tables

**Table 1:** Primers designed for specific detection of SC9-1 vaccine strain by SYBR Green I-based qPCR.

Primers	Sequences (5′-3′)	Product Length
F-SC9-1	GCAGAACCTGCAGGGAATGTA	101 bp
R-SC9-1	TATGGCAGTTAGCGAGCCAG	

**Table 2:** Results of intra-assay and inter-assay repeatability tests.

Plasmid Standards (Copies/μL)	Intra-Assay Repeatability					Inter-Assay Repeatability				
	Ct Value	Mean ($\bar{X}$)	SD (s)	CV (%)	95%CI	Ct Value	Mean ($\bar{X}$)	SD (s)	CV (%)	95%CI
1 × 10^6^	19.21	19.17	0.038	0.1975%	19.07–19.26	19.37	19.37	0.080	0.4130%	19.17–19.57
	19.15					19.45				
	19.14					19.29				
1 × 10^7^	16.27	16.26	0.066	0.4033%	16.10–16.42	16.58	16.53	0.050	0.3046%	16.40–16.65
	16.19					16.48				
	16.32					16.52				
1 × 10^8^	11.24	11.24	0.045	0.4011%	11.13–11.36	11.44	11.54	0.085	0.7372%	11.33–11.75
	11.20					11.57				
	11.29					11.60				

## Data Availability

The original contributions presented in this study are included in the article/Supplementary Materials. Further inquiries can be directed to the corresponding authors.

## Supplementary Materials

The following supporting information can be downloaded at: https://www.mdpi.com/article/10.3390/v18070717/s1, Table S1: Comparison of published qPCR and PCR assays for the identification and differentiation of MDV strains; Table S2: NCBI Nucleotide BLAST results of the SC9-1-specific qPCR target amplicon; Figure S1: Sanger sequencing chromatogram of the REV-LTR chimeric junction fragment amplified from SC9-1 vaccine virus after over 40 serial passages; Data S1: Consensus FASTA nucleotide sequence of the REV-LTR chimeric insertion junction region amplified from SC9-1 vaccine virus after more than 40 serial passages.
