# Supplementary material for: The Development and Evaluation of an SYBR Green I-Based qPCR Assay for Detecting the Marek’s Disease Virus SC9-1 Vaccine Strain
Source: Viruses. 2026 Jun 29;18(7):717. doi: 10.3390/v18070717 (PMC13431530; doi:10.3390/v18070717)
Supplement: Supplementary file 1 [file viruses-18-00717-s001.zip › viruses-4361134-supplementary/Supplementary file.pdf]

Supplementary Materials

The supplementary tables, figure and sequence data provided herein support the strain-specificity and genetic stability of the SYBR Green I-based qPCR assay developed for SC9-1 vaccine strain detection. Table S1 systematically compares all published MDV molecular detection methods; Table S2 summarizes NCBI BLAST homology analysis results of the target REV-LTR chimeric fragment. Figure S1 shows Sanger sequencing chromatograms of serially passaged SC9-1 virus, and Data S1 provides the corresponding consensus FASTA sequence to verify the stable, unique insertion marker of SC9-1.

Table S1. Comparison of published qPCR andPCR assays for the identification and differentiation of MDV strains.

**Assay**	**Detection type**	**Target gene / region**	**Strain specificity**	**Limit of detection**	**Reference**
1	Quantitative-competitive PCR	*gB*	Universal broad-spectrum detection for all MDV serotype 1 strains	Not reported	Reddy et al., 2000[20]
2	TaqMan dual-probe qPCR	*meq* (MDV-1); *sorf1* (HVT)	Separately quantifies MDV serotype 1 and HVT vaccine strain	MDV1: 5 copies/reaction; HVT: 75 copies/reaction	Islam et al., 2006[21]
3	TaqMan single-probe qPCR	*pp38*	Distinguishes CVI988 vaccine strain from virulent wild-type MDV	CVI988: 5.7 copies/reaction; Wild MDV: 9.6 copies/reaction	Baigent et al., 2016[15]
4	Quadruplex TaqMan probe qPCR + Conventional PCR	qPCR: *pp38* (CVI988/wild MDV); *sorf1* (HVT); Conventional PCR: *meq*	qPCR: Differentiates wild MDV, CVI988 and HVT; Conventional PCR: Distinguishes wild MDV, 814 and meq-deleted MDV vaccine strains	CVI988: 10 copies/reaction; Wild MDV: 10 copies/reaction; HVT: 10 copies/reaction	Wu et al., 2023[39]
5	Multiplex conventional PCR	*meq* (MDV-1); *gB* (SB-1); *gB* (HVT)	Separates wild MDV, SB-1 and HVT vaccine strains	1000 copies/reaction	Zhang et al., 2026[18]
6	SYBR Green I-based qPCR (This study)	SC9-1-specific REV-LTR chimeric	Exclusively recognizes only SC9-1 vaccine strain	10 copies/reaction	Present study

Assay	Detection type	Target gene / region	Strain specificity	Limit of detection	Reference
		insertion fragment			

Table S1 Note: As summarized above, all previously reported MDV-specific PCR and qPCR assays (multiplex probe-based qPCR, universal SYBR Green qPCR and end-point PCR) cannot specifically distinguish SC9-1 vaccine strain from other wild MDV strains or commercial MDV vaccines.

Table S2. NCBI Nucleotide BLAST results of the SC9-1-specific qPCR target amplicon

GenBank Accession No.	Organism	Strain/Isolate	Query Coverage (%)	Identity (%)	E-value
JX844666.1	*Gallid alphaherpesvirus 2* (MDV)	GX0101(parental strain of SC9-1)	100	100	2e-43
PV247023.1	*Mardivirus gallidalpha2*	MIS-X	29	100	0.003
L22174.1	*Gallid alphaherpesvirus 2* (MDV)	Regulatory protein gene region	29	100	0.003
PV246975.1	*Mardivirus gallidalpha2*	610A	29	100	0.003
EF526143.1	*Gallid alphaherpesvirus 2* (MDV)	571 IRS/Us junction fragment	29	100	0.003

Table S2 Note: BLAST homology search validated the absolute specificity of the designed amplicon. Only the parent strain GX0101 shared full-length (100% coverage, 100% identical) sequence with our target. All other matched MDV sequences only covered a short 29% conserved genomic segment outside the unique REV-LTR insertion, with no full-length chimeric homologous fragment present in any other MDV strain.

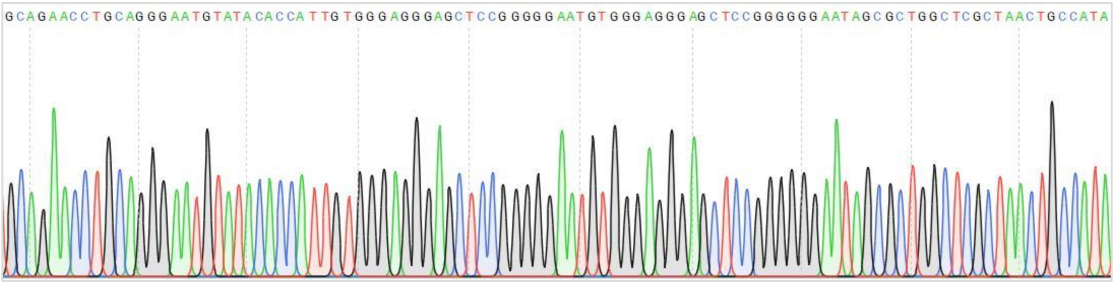

Figure S1. Sanger sequencing chromatogram of the REV-LTR chimeric junction fragment amplified from SC9-1 vaccine virus after over 40 serial passages. The exact passage number cannot be disclosed due to commercial confidentiality. Clear, single peaks without ambiguous superimposed signals indicate high-quality sequencing data. Alignment of the deduced consensus sequence against the parental GX0101 genome (GenBank: JX844666.1) revealed 100% nucleotide identity across the entire target insertion locus, confirming the genetic stability of this strain-specific detection target.

Data S1. Consensus FASTA nucleotide sequence of the REV-LTR chimeric insertion junction region amplified from SC9-1 vaccine virus after more than 40 serial passages. The exact passage number cannot be disclosed due to commercial confidentiality.

SC9-1_HighPassage_over40 passages_REV-LTR_junction

gcagaacctgcagggaatgtatacaccattgtgggagggagctccgggggaatgtgggagggagctccggggggaatagcgctggctcgctaact
gccata
